# PM2.5 downregulates MicroRNA-139-5p and induces EMT in Bronchiolar Epithelium Cells by targeting Notch1

**DOI:** 10.7150/jca.46976

**Published:** 2020-07-29

**Authors:** Yunxia Wang, Yijue Zhong, Cheng Zhang, Jiping Liao, Guangfa Wang

**Affiliations:** 1 Department of Respiratory and Critical Care Medicine, Peking University First Hospital, Beijing, China.; 2 Department of Geriatrics, Jiangsu Provincial Hospital, The First Affiliated Hospital of Nanjing Medical University, Nanjing, Jiangsu, China.

**Keywords:** PM2.5, 16HBE, EMT, Notch1, miR-139-5p

## Abstract

PM2.5 was closely linked to lung cancer worldwide. However, the mechanism involved in PM2.5 induced lung cancer is still largely unknown. In this study, we performed chronic PM2.5 stimulation animal and cells model to investigate the carcinogenetic mechanisms of PM2.5 by targeting EMT through Notch1 signal pathway. Next, we focused on the miRNA involved in PM2.5 induced Notch1 pathway activation. We found chronic PM2.5 could induce EMT event *in vivo* and *in vitro*, while reducing miR-139-5p expression and activating Notch1 pathway meanwhile. And blocking Notch1 signal pathway by specific small molecule inhibitor could reverse PM2.5 induced EMT. Then, overexpression of miR-139-5p downregulated the expression of Notch1 protein in untreated 16HBE cells. Importantly, overexpression of miR-139-5p blocked Notch1 pathway activation and inhibited EMT event in PM2.5 treated cells. These results indicate that PM2.5 induces EMT event through Notch1 signal pathway and miR-139-5p is a novel regulator of PM2.5-induced EMT by targeting Notch1. Our conclusion is that overexpression of miR-139-5p can down-regulate the expression of Notch1 and reverse the occurrence of malignant lung events induced by chronic exposure to PM2.5.

## Introduction

Fine particulate matter (PM2.5) is particulate matter with diameter equal to or less than 2.5 µm and has been closely linked to increased morbidity and mortality of a variety of diseases worldwide, especially lung cancer 1-3. Once the PM2.5 was inhaled, it deposited in lung tissues and diffuses in blood circulation inducing lung and systematic injuries 4. The International Agency for Research on Cancer has formally designated outdoor air pollution in general and ambient particulate matter in particular as human carcinogens 5. Few studies concentrate on the malignant behavior and underlying mechanisms with PM2.5 chronic exposure, which are the most intuitive and representative events for cell fate and deserve greater concerns.

An endless stream of researches indicate that epithelial-mesenchymal transition could accelerate the transition of epithelial cells to mesenchymal cells 6, 7, which serves as a crucial and potential driving force for tumor initiation and progression 8, 9. According to present researches status, cancer cells not only change their properties including motility and invasion, but also gain increased resistance to apoptosis, chemotherapeutic drugs and even develop stem-cell like properties through EMT 10, 11. There are many signal pathways that mediate the emergence of EMT event, such as Wnt, MEK/ERK, TGFβ and Notch 12-14. Notch is an evolutionarily highly conserved signal pathway that plays a key role in lung disease, which contributes to cell proliferation, EMT and chemoresistance 15, 16. NICD serves as the activator of Notch signal pathway, and then translocates NICD cleavage products to the nucleus to activate the expression of Notch target gene Hes1 17.

MicroRNAs (miRNAs) are the important member of endogenous noncoding RNAs family which participates in regulation of cell development, proliferation, differentiation and death 18. Emerging researches suggested that the changes in miRNAs' expression and their posttranscriptional regulator function are linked to many human diseases 19, 20. Researchers have shown that fine particular can alter miRNAs expression in recent years 21, 22. Our pervious study indicated 10 differential miRNAs between PM2.5 exposure and control mice, including miR-146, miR-139 and miR-340 23. However, the specific role of these miRNAs especially in regulating the bronchiolar epithelium cells EMT induced by PM2.5 is still not clear.

In the present study, we established the hypothesis that PM2.5 induces EMT in bronchiolar epithelium cells. Next, we focused on the related regulatory mechanisms and figured out that PM2.5 regulates the expression of miR-139-5p and induces bronchiolar epithelium cells EMT by activating Notch1.

## Materials and Methods

### PM2.5 collection and preparation

The PM2.5 was collected by a special machine called high volume sampler system (Staplex PM2.5 SSI, USA) in December 2016 at Peking University First Hospital, which located in the central area of Beijing. The collection method and component analysis report of PM2.5 had been described in previous published literature 23. The PM2.5 suspension was obtained by thoroughly mixing the particulate matter and normal saline to treat mice, or culture medium with 1% fetal bovine serum under sonication to stimulate cells.

### Cell culture and treatment

The human bronchial epithelial cells 16HBE was cultured in Dulbecco's modified Eagle's medium (DMEM, Sigma-Aldrich, USA) supplemented with 10% fetal bovine serum (Gibco, USA), 100U/ml penicillin and 100 μg/ml streptomycin (Gibco, USA) and maintained at 37°C in a saturated humidity atmosphere containing 5%CO_2_. 16HBE cells were treated with different concentrations of PM2.5 (0, 25, 50, 100 µg/ml) for 24-48 hours per passage, and this process was continued for five passages to mimic chronic exposure. MiR-139-5p mimic and its negative control, anti-miR-139-5p and anti-miR-139-5p negative control were purchased from RIBOBIO Company (Guangzhou, China). MiR-139-5p, anti-miR-139-5p and corresponding control microRNAs were complexed respectively with ribo FECT™ CP Transfection Kit (RIBOBIO, Guangzhou, China) according to the manufacturer's instructions. 16HBE cells were transfected with miR-139-5p mimic (50 µM), miR-139-5p inhibitor (100 µM) and corresponding negative control for 12h followed by PM2.5 (100 µg/ml) per passage, eventually to continue for five passages.

### Cell treatment with inhibitor

16HBE cells were pretreatment with Notch1 inhibitor named RO4929097 (Selleck, USA) for about 2 hours before the first passage. In the other four passages, RO4929097 was mixed into the culture medium with or without PM2.5 to make sure the final concentration of RO4929097 and PM2.5 solution were 10 uM and 100 ug/mL, respectively.

### Animal

Thirty-two adult BALB/c mice (male, 8 weeks old, 25±2g) were purchased from Beijing Vital River Laboratories (license number: SCXK (JING) 2016-0011) and quarantined. There after they were raised in the animal room conditions for at least one week before any operation. The animals were housed in the specific pathogen-free environment with room temperature (23-25°C), relative humidity (40%-70%) and 12h light/dark cycles. The study conformed to the principles for laboratory animal research outlined by the Animal Welfare Act and approved by Laboratory Animal Ethics Committee of Peking University First Hospital (permit no. J201609).

### Intratracheal instillation of PM2.5 sample to mice and sample collection

The mice were randomly and equally divided into control and PM2.5-treated groups: low dose group (2.5 mg/kg), middle dose group (10 mg/kg), high dose group (20 mg/kg). Mice were anesthetized by intraperitoneal injection of 5% chloraldurate, the PM2.5-treated groups were intratracheally instillized PM2.5 suspension with the dosage of 2.5, 10 or 20 mg/kg in 50 μl normal saline respectively (n=8 for each dosage), every 3 days for over 90 days to mimic chronic PM2.5 exposure. The control group was treated with an equal volume normal saline (n=8) in the same manner. The animals were euthanized on the 90th day after intratracheal instillation. After 90 days of sacrification, bilateral lung was extracted for further study. After that, the middle zone of the left lung was cut off sagittally for pathological examination. The right lung was stored in liquid nitrogen for RNA and protein extraction and quantification.

### Pathological examination of lung tissue

Lung tissue near hilar region of left lung was taken and processed for light microscopy. For light microscopy, the lung tissue was fixed in 4% paraformaldehyde solution. After paraffin embedding, 4 μm sections were cut and stained with hematoxylin and eosin to reveal lung tissue pathology. The sections were evaluated by light microscopy using a microscope (DP-72, Olympus, Japan), and semi-quantitative analysis of immunohistochemistry were carried out with Image-Pro Plus 6.0 software (Media Cybernetics, USA).

### Transmission electron microscope (TEM)

Lung tissue of right lung was taken and cut into millet grain size particles, then processed for transmission electron microscope. Subsequently, samples of lung were fixed in 3% glutaraldehyde, Dulbecco's PBS, and 0.5% osmiumtetroxide/0.025M potassium hexacyanoferrate(III) solution, followed by incubation in uranylacetate/70% ethanol overnight. Samples were then dehydrated in ascending ethanol solutions starting from 80% ethanol and embedded in epoxy resin (EPON) blocks. Subsequently, blocks were cut in smaller blocks of ~1 mm diameter. Ultrathin sections (80 nm) were taken with a diamond blade from EPON blocks (Reichert ULTRACUT, Leica, Wetzlar, Germany), mounted on a 200 mesh hexagonal platinum grid and further contrasted in a 0.2% lead citrate/0.1 M sodium hydroxide solution for 20 min. Photos were taken with the MORADA camera (Olympus Soft Imaging System, Mu¨nster, Germany) using a transmission electron microscope (FEI Thermo Fisher Scientific, Munich, Germany) with magnifications as depicted.

### Quantitative assessment of mRNA and miRNA Expression

TRIZOL reagent (Invitrogen Life Technologies, USA) was used to extract total RNA. 1µg total RNA quantified by Nanodrop 2000 (Thermo Fisher Scientific, USA) from each sample was reversely transcribed to 20 µl complementary DNA (cDNA) according to the instruction of RevertAid First Strand cDNA Synthesis Kit (Thermo Fisher Scientific, USA). For miRNAs, reverse transcription of 600ng total RNA was conducted by using Mir-XTM miRNA First Strand Synthesis Kit (Clontech Laboratories, Inc. USA). The primers of mRNA and miRNA specific primers were purchased from Sango Biotech Company (Shanghai, China). The primers used for qRT-PCR were shown in Table 1. The forward and reverse primer of U6 were included in the Mir-XTM miRNA First Strand Synthesis Kit. qRT-PCR was carried out in a 20 µl reaction system containing 1 µl cDNA and 10 µl POWER SYBR® Green Master Mix (Thermo Fisher Scientific, USA) on Step One Plus Real-Time PCR System (7500, Applied Biosystems, USA). The relative expression of mRNA or miRNA was calculated by 2-ΔΔCT method as described elsewhere and normalized to the expression of β-actin or U6 respectively.

### Luciferase analysis

According to the binding site on Notch1 mRNA 3'-untranslated region (3'-UTR), a wild-type (wt) Notch1-3'-UTR gene or a mutated (mut) Notch1-3'-UTR gene was constructed and cloned into the pMIR-REPORT miRNA expression reporter vector (Obio Technology Corp, Shanghai, China). The HEK293T cells (National Infrastructure of Cell Line Resource, Shanghai, China) were transfected with empty vector, Notch1-3'-UTR-wt vector and Notch1-3'-UTR-mut vector with miR-139-5p mimic or scramble control. After 48 h, the transfected cells were analyzed by Dual-Luciferase Reporter Assay System (Promega Corporation, Fitchburg, WI, USA).

### Western blot for protein

The proteins expressions were detected by western blotting. The total proteins were extracted with a precooling RIPA lysis buffer containing 1% phosphatase inhibitor cocktail. The cell or lung tissue lysate was centrifuged at 12,000 rpm for 15 min at 4°C; the supernatant fluid was subsequently collected. The protein content was determined using the Pierce BCA protein assay kit according to the manufacturer's instructions. The proteins were separated through sodium dodecyl sulfate-polyacrylamide gel electrophoresis. The protein samples were transferred to NC membranes and blocked with 5% nonfat milk. Thereafter, the membranes were incubated with E-cadherin (1:1000, abcam, USA), Vimentin (1:1000, Wanleibio, China), Notch1 (1:800, PL Laboratories, Canada), Hes1 (1:1000, ABclonal, USA) and β-actin (1:1000, Zsbio, China) antibody overnight on a shaker at 4°C, followed by the appropriate secondary antibody for 1h at room temperature. The proteins were quantified and visualized using the Syngene GeneGenius (GBOX-CHEMI-XT4, SYNGENE, USA).

### Statistical analysis

Statistical analyses were performed using the SPSS20.0 system and GraphPad Prism 5.0 software (GraphPad Software Inc., USA). Statistical differences among experimental groups were evaluated with one-way ANOVA, followed by LSD multiple comparison post-hoc test. For the data of heterogeneity of variance, independent samples nonparametric tests with Kruskal-Wallis H Test were used. A *P* value of <0.05 was considered as statistically significant. Difference significance among groups was assessed as * *p* < 0.05; ** *p* < 0.01; *** *p* < 0.001.

## Results

### Histopathological and ultrastructural changes in lung treatment with PM2.5

**Figure 1:**
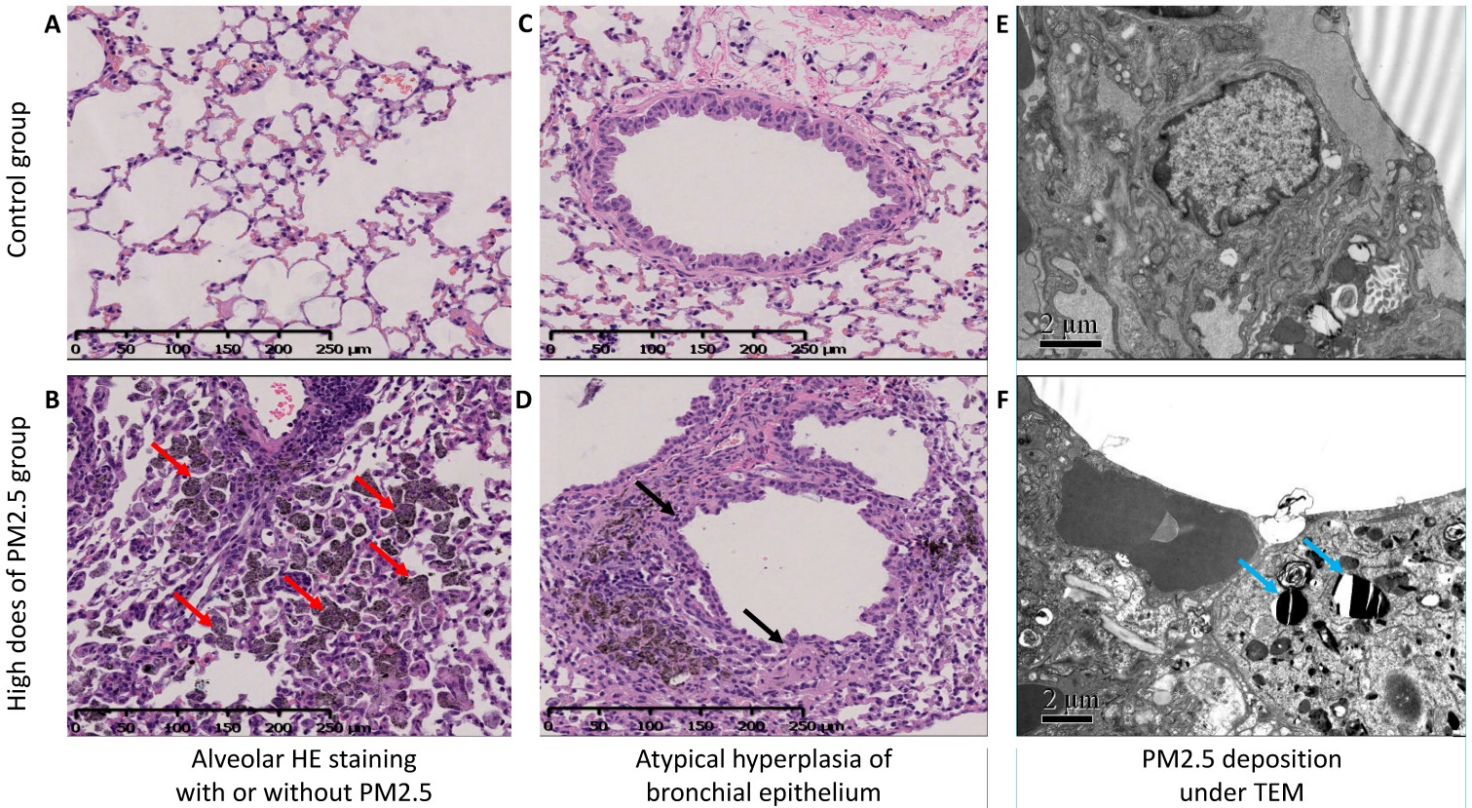
** HE stain and TEM in lung tissue.** (**A and B**) The HE stained alveolar tissue in a lung section, A was the control group, and C was the high dose group. The red arrow showed plentifully infiltration of macrophages with particulate matter deposition and alveolar cavity narrowed and solid. (**C and D**) The atypical hyperplasia of bronchiolar epithelium of (C) the control group and (D) the part of glands papillary hyperplasia with some luminal glands crowded and disordered by black arrow. (**E and F**) Transmission electron microscope for (E) the control group and (F) the high dose group. The blue arrow showed the deposition of PM2.5 in the cytoplasmic lysosome as were rod-like, crystal-like and layered and the changes of cell morphology.

Hematoxylin-eosin (H-E) stained lung sections in high dose group of PM2.5 exposure mice, it showed plentifully infiltration of macrophages with particulate matter deposition (Figure 1B), and atypical hyperplasia of bronchiolar epithelium and part of glands papillary hyperplasia with some luminal glands crowded and disordered (Figure 1D). In the transmission electron microscope, we could find the ultrastructural changes of the cells. And among them, the most important of which was the deposition of PM2.5 in the cytoplasmic lysosome as were rod-like, crystal-like and layered and the changes of cell morphology (Figure 1F).

### Chronic PM2.5 exposure stimulated EMT of bronchial epithelial cells *in vivo* and *in vitro*

**Figure 2:**
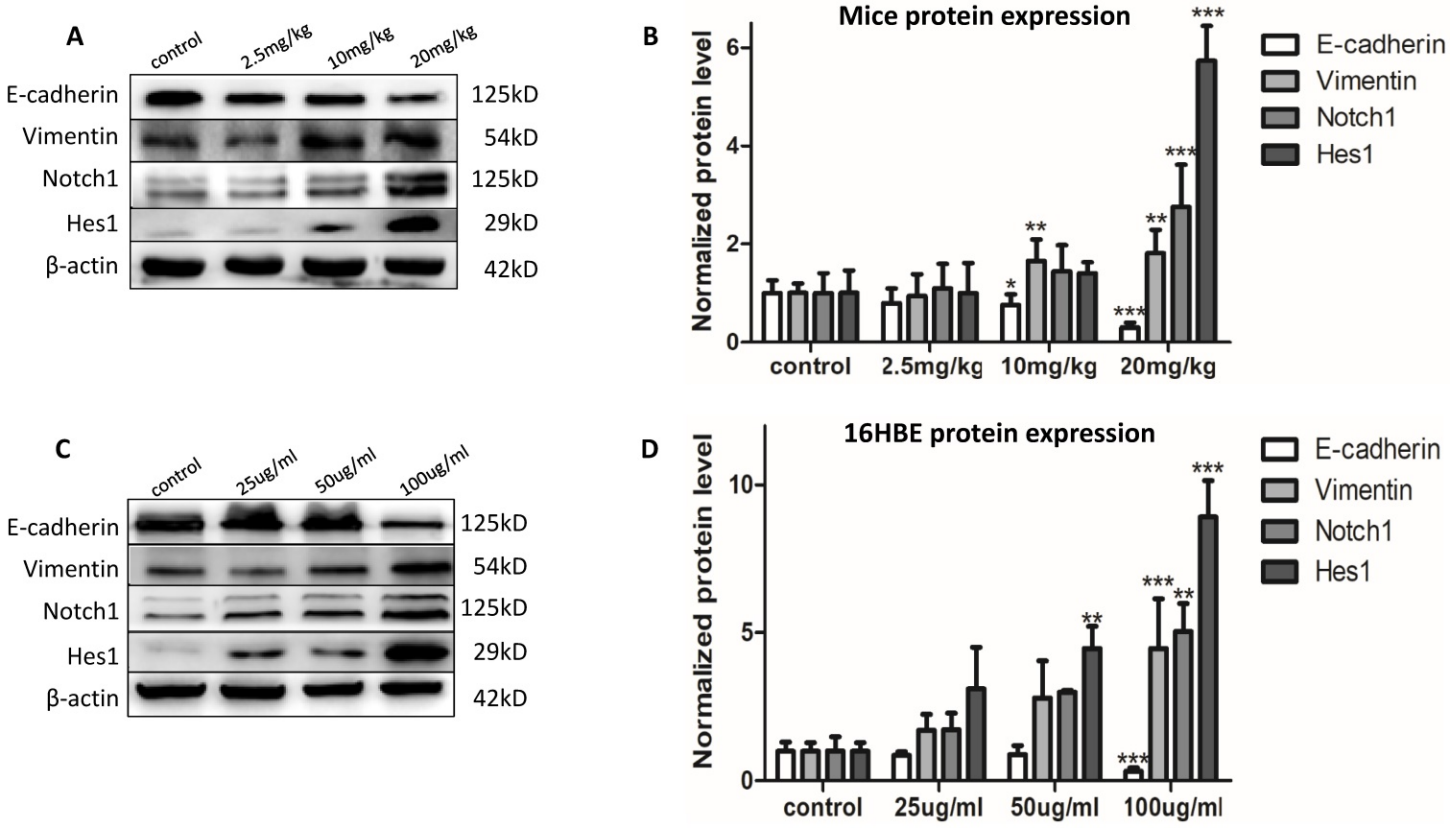
** The EMT event and Notch1 signal pathway *in vivo* and *in vitro*.** (**A and B**) EMT and Notch1 signal pathway event in mice (n=3). (**C and D**) EMT event and Notch1 signal pathway in 16HBE cells (n=3). *P < 0.05, **P < 0.01, ***P < 0.00, compared with control.

The expression of the EMT event markers was determined to evaluate the EMT effects. After chronic PM2.5 exposure, the protein expression of Vimentin increased in middle and high dose group; and E-cadherin decreased in the two groups (Figure 2A). Next, we have determined the atypical hyperplasia of bronchiolar epithelium in Figure 1F. To determine whether chronic PM2.5 exposure could induce EMT event in 16HBE cells, EMT event, the developmental process of malignant transformation, was further determined. After exposure for five passages, we found the alterations of protein expression as decreased protein levels for E-cadherin, and increased protein levels for Vimentin (Figure 2C). These results indicated that chronic PM2.5 exposure caused EMT event. These results above indicated that chronic PM2.5 exposure could cause obvious changes in protein expression of EMT markers, suggesting the EMT event *in vivo* and *in vitro* caused by PM2.5.

### Chronic PM2.5 exposure activated Notch signal pathway of lung *in vivo* and *in vitro*

After confirming the EMT event induced by PM2.5 *in vivo* and *in vitro*, we were eager to figure out the signal pathway involved into this process. We detected the protein expression of Notch1 and Hes1 in mice and 16HBE cells after chronic PM2.5 stimulation by western blot. The results showed that PM2.5 activated Notch1 signal pathway manifesting as increased protein expression of Notch1 and Hes1 in Figure 2A and C.

### Inhibiting Notch1 pathway could block EMT event induced by chronic PM2.5 exposure in 16HBE cells

**Figure 3:**
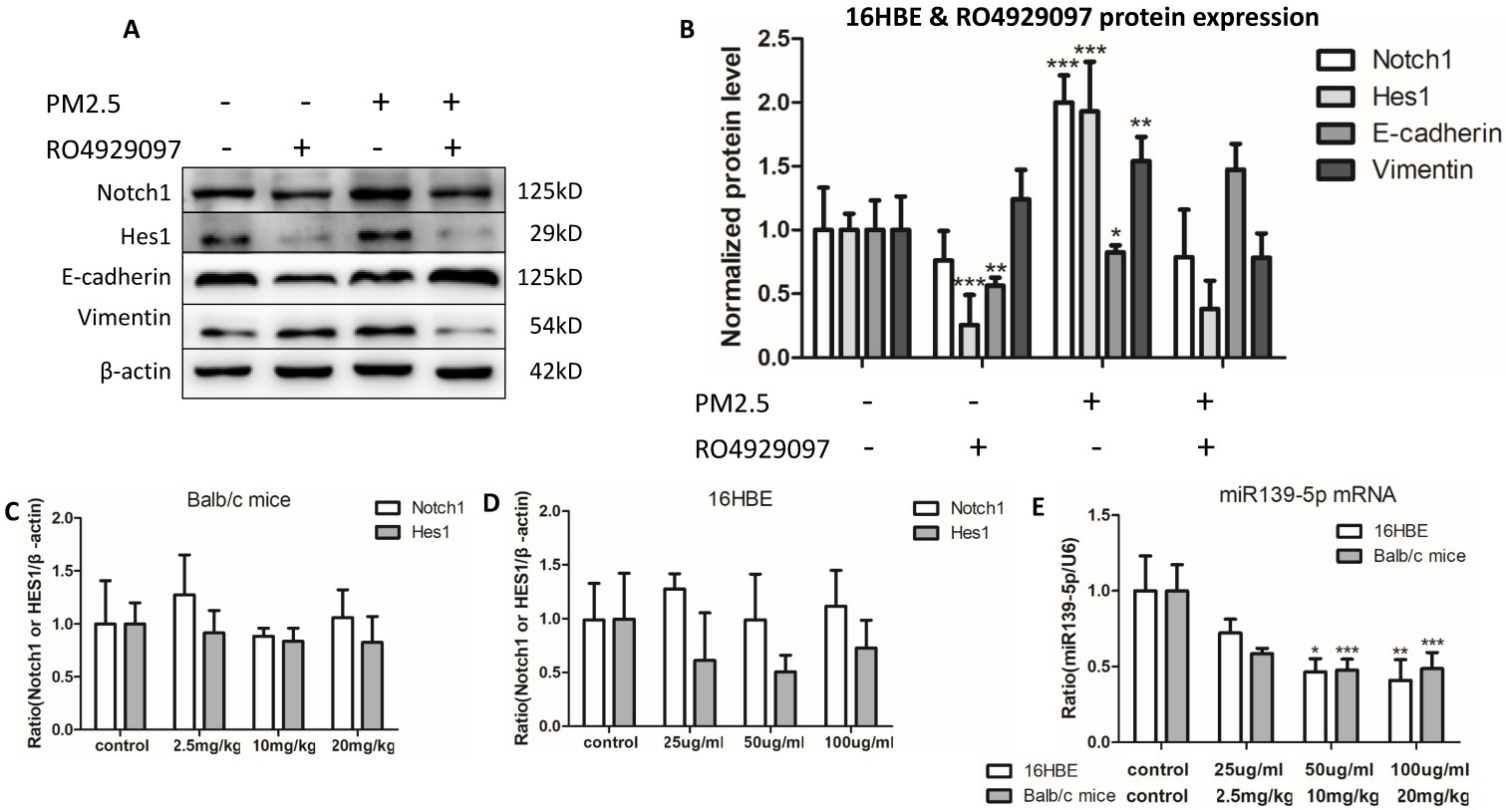
** Inhibiting Notch1 pathway block EMT event induced by chronic PM2.5 exposure and the mRNA expression of Notch1 and Hes1 and miR-139-5p *in vivo* and *in vitro*.** (**A and B**) Relative protein expressions of Notch1 signal pathway markers and EMT markers (n=3). (**C**) mRNA expression of Notch1 and Hes1 *in vivo* (n=3). (**D**) mRNA expression of Notch1 and Hes1 *in vitro* (n=3). (**E**) miR-139-5p expression *in vivo* and *in vitro* (n=3). *P < 0.05, **P < 0.01, ***P < 0.00, compared with control.

PM2.5 induced EMT event have been proved *in vivo* and *in vitro* above, while signal pathway molecular Notch1 and Hes1 also increased, and we have demonstrated that inhibition of Notch1 pathway could reverse PM2.5-induced EMT in A549 and BEAS-2B cell lines. So could it also reverse PM2.5-induced EMT through blocking up Notch1 signal pathway in 16HBE cells? We selected RO4929097 as the Notch1 inhibitor. To investigate the effect of RO4929097 on the EMT, we performed the protein levels of Notch1, Hes1, E-cadherin and Vimentin using western blot in 16HBE cells. Results indicated that RO4929097 inhibited the activation of Notch1 signal pathway and reversed the EMT process, manifesting as decreasing the protein levels of Notch, Hes1, Vimentin and up-regulating the expression of E-cadherin with five passages chronic PM2.5 exposure (Figure 3A).

### PM2.5 decreased the expression of miR-139-5p and Notch1 was a potential target of miR-139-5p

**Figure 4:**
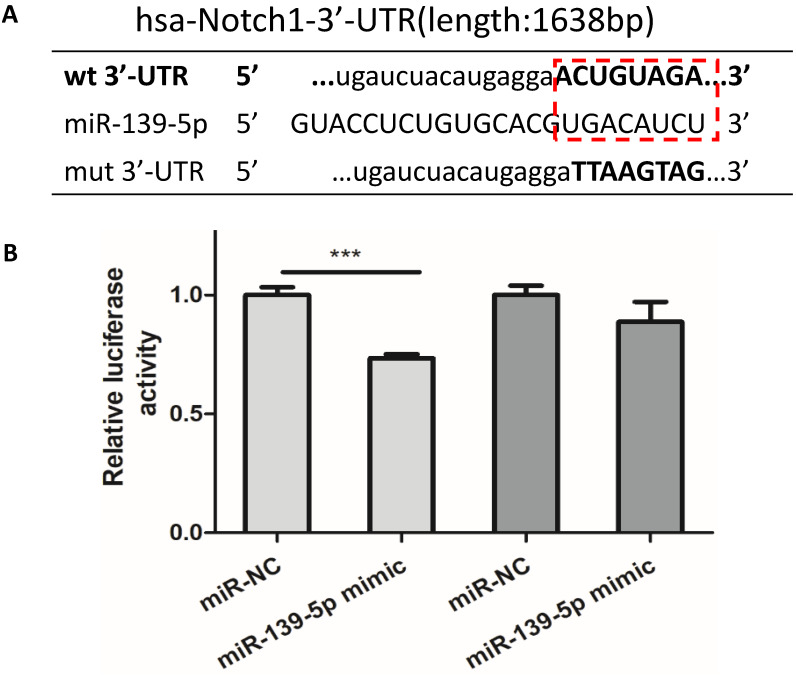
** Notch1 was a potential target of miR-139-5p.** (A) Wild-type and mutant binding sites of miR-139-5p in the 3′-UTR of Notch1. (B) Luciferase analysis. The results showed that miR-139-5p mimics decreased the fluorescence intensity in cells transfected with Notch1-3′-UTR-wt but did not change the fluorescence intensity in cells transfected with Notch1-3′-UTR-mut. *P < 0.05, **P < 0.01, ***P < 0.00, compared with control.

In order to observe the expression of miR-139-5p in PM2.5 treated 16HBE cells, we detected the expression of miR-139-5p by qRT-PCR. Compared with control group, the expression of miR-139-5p showed a significant decrease in 50 and 100µg/ml PM2.5 exposure group (Figure 3E). In order to explore the mechanism of miR-139-5p in PM2.5 stimulated 16HBE cells, we discovered that Notch1 was a potential regulatory targets of miR-139-5p predicted in three bioinformatics databases (TargetScan, miRanda and miRWalk). As shown above in Figure 2 and 3, the protein expression but not the mRNA expression of Notch1 was increased after PM2.5 stimulation. This indicated that miR-139-5p might decrease the expression of Notch1 through post-transcriptional regulation. The putative target sequence was located in the 1510-1517nt of the 3'-UTR of human Notch1 mRNA. To verify the possible binding sites, the luciferase analysis was used to identify the site of miR-139-5p and Notch1. The Notch1 mRNA containing the wild-type (wt) putative binding site or mutated (mut) binding site of miR-139-5p were constructed (as shown in Figure 4A) and clone into the luciferase expressing pMIR vector. Notch1-3'-UTR-wt vector or Notch1-3'-UTR-mut vector was co-transfected with hsa-miR-139-5p mimic or control (miR-NC) into HEK293T cells respectively. The results indicated that the relative luciferase activity decreased significantly in HEK293T cells transfected with Notch1-3'-UTR-wt vector, but no significant change in cells transfected with Notch1-3'-UTR-mut (Figure 4B). Above all, the result confirmed that miR-139-5p directly negatively regulated Notch1.

### Overexpression of miR-139-5p directly downregulated the expression of Notch1 protein

**Figure 5:**
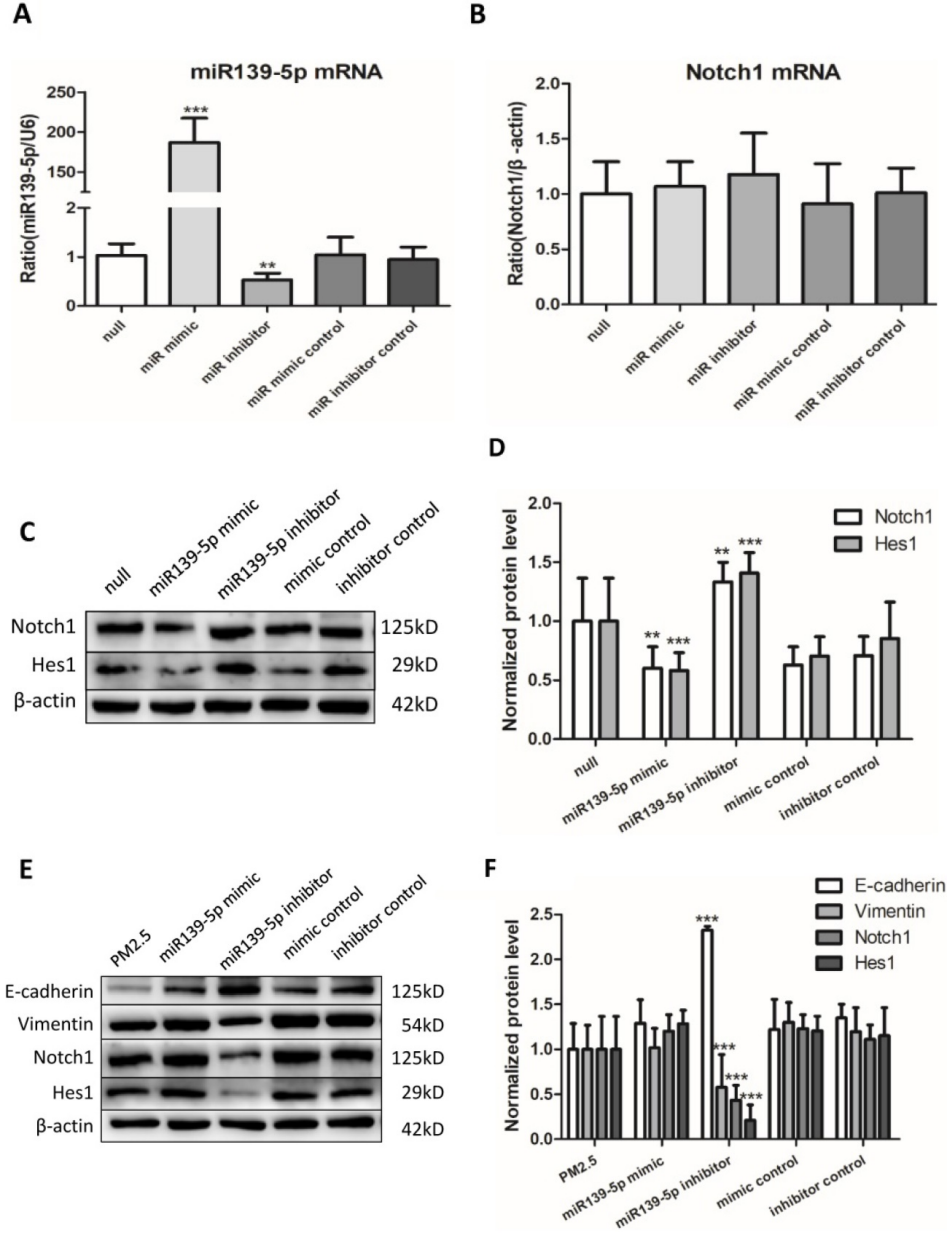
** Inhibiting miR-139-5p downregulated the expression of Notch1 protein and reversed EMT event in PM2.5 treated 16HBE cells.** (**A and B**) Relative expression of miR-139-5p normalized against the U6 endogenous control and Notch1 mRNA normalized against the β-actin endogenous control in untreated 16HBE transfected with miR-139-5p mimic, inhibitor or scrambled controls (n=3). (**C and D**) Overexpression of miR-139-5p directly downregulated the expression of Notch1 protein without PM2.5 exposure in 16HBE cells (n=3). (**E and F**) Overexpression miR-139-5p downregulated the expression of Notch1 protein and reversed EMT event in PM2.5 treated 16HBE cells (n=3). *P < 0.05, **P < 0.01, ***P < 0.00, compared with control.

To continue explore the potential regulation relationship between miR-139-5p and Notch1, untreated 16HBE cells were transfected with miR-139-5p mimic, inhibitor and controls for 24h. The efficiency of transfection and the expression of Notch1 mRNA were detected by qRT-PCR (Figure 5A and B). The expression of Notch1 was detected by western blotting (Figure 5C). The Notch1 mRNA expression showed no significant difference, while Notch1 protein increased significantly after inhibition of miR-139-5p and decreased significantly after overexpression of miR-139-5p. These data showed that miR-139-5p mimic downregulated the expression of Notch1 protein in 16HBE cells without PM2.5 stimulation.

### Overexpression miR-139-5p directly down-regulated the expression of Notch1 protein and reversed EMT event in PM2.5 treated 16HBE cells

Last but not least, to discover whether miR-139-5p could regulate EMT event in PM2.5 stimulated 16HBE cells, cells were transfected with miR-139-5p mimic (50 µM), miR-139-5p inhibitor (100 µM) and corresponding negative control for 12h followed by PM2.5 (100 µg/ml) per passage, eventually to continue for five passages. Western blot results showed the Notch1 protein expression decreased significantly in 16HBE cells treated with inhibitor compared with PM2.5 treated group. Overexpression of miR-139-5p significantly increased E-cadherin and decreased Vimentin, Notch1 and Hes1 in PM2.5 treated 16HBE cells. On the contrary, inhibition of miR-139-5p significantly increased the Notch1 signal pathway and EMT event protein markers (Figure 5E). The results demonstrated that increased the expression of miR-139-5p could down-regulate Notch1 protein and suppressed EMT event in PM2.5 treated 16HBE cells.

## Discussion

China suffers from serious environmental pollution as a developing country, blamed on the rapid period of urbanization and industrialization. Among the environmental pollution, the most serious and widespread one is air pollution and it can't be eliminated or significantly reduced in short term 24. PM2.5 is a complex mixture of small particles and liquid droplets in the air. Our previous studies indicated that PM2.5 contained high concentration of endotoxin up to 20.8 ng/m^3^ and toxic heavy metals such as Cu, Zn, Al, Mn and Pb 23, 25, which was similar to others 26-28. And lung cancer is a crucial disease with high morbidity and mortality all around the world. PM2.5 exposure is associated with lung cancer in epidemiological researches 29-31.

In our study, high dose of chronic PM2.5 stimulation induced atypical hyperplasia of bronchiolar epithelium in mice, while it was a morphological change of precancerous lesions through EMT event. EMT, an event characterized as epithelial properties loss and mesenchymal properties acquisition, is considered as a critical step in tumor initiation, progression 8, 32 as well as invasion and metastasis 7. Our observations indicate the involvement of EMT in PM2.5-induced malignant transformation *in vivo*, which may provide biological explanations and compelling support for the epidemiological association and IARC's evaluation. In addition to the part of the *in vivo*, we also want to figure out whether PM2.5 could cause EMT of bronchial epithelium cells *in vitro*. As we have confirmed that PM2.5 could induce EMT event in A549 and BEAS-2B cell lines as alveolar cells 33, this time we valued the EMT event in 16HBE cells as bronchial epithelium cell due to the atypical hyperplasia in mice. We found the same conclusion consistent with the experiment *in vivo*: PM2.5 could induce EMT.

In addition to the phenotypic changes induced by PM2.5 *in vivo* and *in vitro*, the related pathogenesis is also very important in this study. Notch signaling pathway is highly conserved that regulates a vital role in proliferation, stem cell maintenance, cell fate specification, differentiation, and homeostasis of multicellular organism and implicates angiogenesis, involved in many diseases 34-36. Among the Notch families, Notch1 has recently been linked to the pathogenesis of EMT in lung cancer 37. During this study, we have verified the EMT event induced by chronic PM2.5 exposure, so we want to confirm that whether PM2.5 stimulation could alter the Notch signal pathway markers expression. Eventually, after chronic PM2.5 exposure, Notch signal pathway was activated demonstrated as increased expression of Notch1 and Hes1 *in vivo* and *in vitro*. To prove that Notch1 signal pathway was indeed involved in PM2.5-induced EMT in bronchial epithelium cells, we used selected inhibitor RO4929097 to block Notch1 signal pathway, and EMT event was also been inhibited further, which meant chronic PM2.5 exposure induced 16HBE EMT through Notch1 signal pathway. Overall, blocking-up Notch1 could suppress EMT, which meant Notch1 signal pathway had an intimate connection with malignant behaviors of 16HBE cells.

MiRNAs are acknowledged as key molecules in post-transcriptional modification. They can bind directly to the 3'-UTR of target mRNA leading to mRNA degradation or translation suppression 38. On account of the stable expression and the conservative role, miRNAs have been considered to have therapeutic potential and became a research focus in recent years 39-41. Our research team has indicated several miRNAs upregulated including miR-139-5p in lung tissues of mice acute exposed to PM2.5 and involved in regulating Th1/Th2 balance 23. Among these miRNAs, miR-139-5p has been indicated to be involved in different cancers by Notch 42-45. However, the role of miR-139-5p in PM2.5 exposed bronchial epithelium cells has not been elucidated. In this study, we discovered that miR-139-5p directly targeted Notch1 which could regulate PM2.5-induced EMT as mentioned above. Our study demonstrated that PM2.5 reduced miR-139-5p then up-regulated Notch1 protein in mice and 16HBE cells. Not only that, inhibition of miR-139-5p up-regulated Notch1 protein and induced EMT event in PM2.5 treated 16HBE cells.

In conclusion, our results revealed that the chronic PM2.5 exposure induced the acquisition of EMT *in vivo* and *in vitro*, and Notch1 signal pathway involved in PM2.5-induced EMT as blocking Notch1 could negatively regulate EMT. Most important of all, PM2.5 reduced the expression of miR-139-5p to up-regulate Notch1 protein expression, further induced EMT event in bronchial epithelial cells. This suggested that miR-139-5p might be a potential therapeutic target of EMT event in lung cancer induced by PM2.5.

## Figures and Tables

**Table 1:** The primers used for qRT-PCR

Species	Gene name	forward	reverse
Mouse	miR-139-5p	TCTACAGTGCACGTGTCTCCAGT	
	Notch1	GATGGCCTCAATGGGTACAAG	TCGTTGTTGTTGATGTCACAGT
	Hes1	CCAGCCAGTGTCAACACGA	AATGCCGGGAGCTATCTTTCT
	β-actin	GCTTCTTTGCAGCTCCTTCGT	AGCGCAGCGATATCGTCATC
Human	miR-139-5p	ACACTCCAGCTGGGTCTACAGTGCACGTG	
	Notch1	CGGGTCCACCAGT TTGAATG	GTTGTATTGGTTCGGCACCAT
	Hes1	AACCAAAGACAGCATCTGAGCAC	TGTAGACCATGTAGTTGAGGTCA
	β-actin	CACCATGAAGATCAAGATCATTGC	GGCCGGACTCATCGTACTCCTGC
